# Oral and gut microbial biomarkers of susceptibility to respiratory tract infection in adults: A feasibility study

**DOI:** 10.1016/j.heliyon.2023.e18610

**Published:** 2023-07-28

**Authors:** Claire A. Woodall, Ashley Hammond, David Cleary, Andrew Preston, Peter Muir, Ben Pascoe, Samuel K. Sheppard, Alastair D. Hay

**Affiliations:** a School of Cellular and Molecular Medicine, Bristol Medical School, University of Bristol, Bristol, UK; b Centre for Academic Primary Care, Population Health Science, Bristol Medical School, University of Bristol, Bristol, UK; c Institute of Microbiology and Infection, College of Medical and Dental Sciences, University of Birmingham, Edgbaston, Birmingham, UK; d The Milner Centre for Evolution and Department of Biology and Biochemistry, University of Bath, Bath, UK; e Public Health England, Southwest Regional Laboratory, National Infection Service, Southmead Hospital, Bristol, UK; f Department of Biology, University of Oxford, Oxford, UK

**Keywords:** Respiratory tract infection, Microbial biomarkers, Longitudinal, Community, Feasibility

## Abstract

We conducted a feasibility cohort study which aimed to recruit and retain adults from the community to collect saliva (oral) and stool (gut) samples at three time points, at the start of the study (baseline), during a respiratory tract infection (RTI) and post-RTI. Community RTIs place a huge burden on health care services, and a non-invasive microbial diagnostic tool to predict the most vulnerable to respiratory infection would be ideal. To this aim, we analysed oral-gut baseline samples comparing those who reported RTI symptoms to those who remained healthy throughout the study for microbial biomarkers of respiratory susceptibility. Amplicon sequence variants (ASV) were identified by 16S sequence profiling to reveal oral-gut microbes. Reverse transcriptase-polymerase chain reaction (RT-PCR) was applied to target common respiratory microbes. Two general practices were recruited, and the participant recruitment rate was 1.3%. A total of 40 adult participants were retained, of which 19 acquired an RTI whereas 21 remained healthy. In healthy baseline oral and gut samples, ASVs from participants with RTI symptoms compared to those who remained healthy were similar with a high relative abundance of *Streptococcus* sp., and *Blautia* sp., respectively. Linear discriminant analysis effect size (LEfSe) revealed baseline oral microbes differed, indicating participants who suffered RTI symptoms had enhanced *Streptococcus sobrinus* and *Megamonas* sp.*,* and depletion of *Lactobacillus salivarius*, Synergistetes, Verrucomicrobia and Dethiosulfovibrio. Furthermore, a random forest model ranked *Streptococcus* (4.13) as the highest mean decrease in accuracy (MDA) and RT-PCR showed a higher level of carriage of coagulase-negative *Staphylococcus*. Baseline core gut microbes were similar in both participant groups whereas LEfSe analysis revealed enhanced *Veillonella*, Rikenellaceae, *Enhydobacteria*, *Eggerthella* and *Xanthomonsdales* and depleted *Desulfobulbus* and *Coprobacillus*. *Sutterella* (4.73) had a high MDA value. Overall, we demonstrated the feasibility of recruiting and retaining adult participants from the community to provide multiple biological samples for microbial profiling. Our analyses identified potential oral-gut microbial biomarkers of respiratory infection susceptibility in otherwise healthy participants.

## 1 Introduction

Respiratory tract infections (RTIs) are the most common infections seen in primary care, and the single greatest contributor to the overall burden of disease worldwide [1]. In the UK, respiratory illness costs the National Health Service (NHS) £11 billion per year [2]. Seasonal community-acquired viral RTIs such as common colds, bronchitis, and influenza-like illness (ILI) are common in adults, who may experience multiple infections per year. COVID-19 disease caused by severe acute respiratory syndrome coronavirus-2 (SARS-CoV-2) is now classified as a seasonal RTI and has resulted in an increase burden on frontline NHS services amounting to £4-5bn a year [3]. One way to reduce the burden of RTIs on global health services is early identification of those individuals who are more likely to acquire a RTI so that protective measures can be implemented in a timely manner.

The human microbiome is an important moderator of health and disease. The oral cavity and the gut are the two largest human microbial ecosystems and are connected at a shared mucosal surface [4]. This linked oral-gut niche contains a dynamic microbial balance where beneficial microbes confer protection against pathogens through competition for nutrients, bacteriocin production and biosynthesis of molecules that indirectly induce local immune responses [[5], [6], [7], [8]]. Insights into microbial signatures at both the oral-gut may offer insight into susceptibility to RTI [[9], [10], [11]]. Susceptibility or risk biomarkers are associated with an increased (or on occasion decreased) chance of developing a disease or infection, from a clinical standpoint, in those individuals who otherwise appear healthy [12]. They can be detected years before the appearance of clinical signs and symptoms, usually from baseline samples [13,14]. Recently, the emerging picture of the role of the human microbiome in disease states has led to interest in potential microbial risk biomarkers of disease, including infection. For example, several studies have described gut microbial biomarkers inferring differential susceptibility to SARS-CoV-2 infection [14,15].

Most microbial biomarker studies usually target a single host niche, few cohort studies simultaneously test two or more host sites. Emerging research has tracked both the upper respiratory tract and gut microbiota changing and then recovering during a COVID-19 infection episode in adults and children [[16], [17], [18]]. A further study in infants demonstrated that microbial networks over three sites, the oral cavity, nasopharynx, and gut are connected and associated with the risk of respiratory infection acquisition [19]. Although microbiota are immature and unstable in infancy [20], these studies are encouraging as they highlight the potential for the microbiota to influence RTI acquisition. However, currently there is a lack of substantial multi-microbiota cohort studies for any respiratory pathogen that describes with certainty susceptibility biomarkers to respiratory infection from healthy baseline samples, particularly including participants from the community, i.e., participants regarded as generally healthy, without underlying health issues or predispositions to RTIs, who represent the majority of seasonal RTIs presenting to general practice.

Here, our primary objective was to test the feasibility of recruiting and retaining adults from the community during the winter season, to complete a self-reported questionnaire and to collect saliva and stool samples at the start of the study (A), during a RTI (B) and recovery from RTI symptoms or end of study samples (C) for microbial analysis. During the study we anticipated that some participants would develop RTI symptoms (RTI-S) whereas other may remain healthy (no-RTI) and hypothesised that differences in the microbial profiles between these two groups will indicate susceptibility to respiratory infection. The secondary objective was to characterise oral-gut microbes by 16S microbial profiling at all time points and to apply biomarker analysis to identify putative microbial biomarkers of respiratory infection susceptibility in RTI-S participants compared to those who remained healthy, from baseline samples.

We demonstrate the feasibility of this approach. Additionally, as part of our secondary objective, we identified putative oral-gut microbial biomarkers for RTI susceptibility. Microbial biomarkers of susceptibility for community based acute RTIs are underrepresented in the literature but can provide valuable insight into the early identification of those who may be vulnerable to respiratory infection. Our work should encourage other larger studies aimed at predicting respiratory infection in otherwise healthy participants in a primary care setting by multi-microbiota biomarker application.

## 2 Methods

### 2.1 Ethical declaration

The study was approved by the Southwest – Central Bristol Research Ethics Committee (REC Reference 19/SW/0167) on October 2, 2019. NHS Health Research Authority approval was granted on October 22, 2019. Our ethics proposal stated this study will explore the influence of the human microbiome in acquisition of respiratory infections in the community and information obtained will be used to inform a larger cohort study including the calculation of a longitudinal microbial power calculation.

### 2.2 Study design

**Fig. 1:**
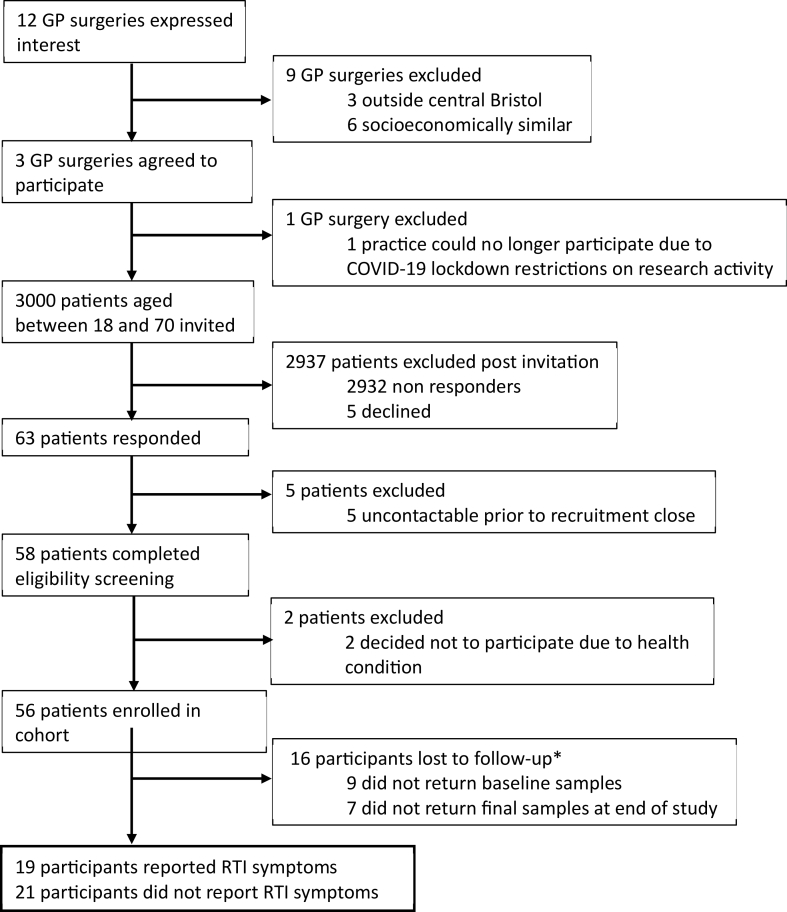
Participant recruitment flowchart.

**Fig. 2:**
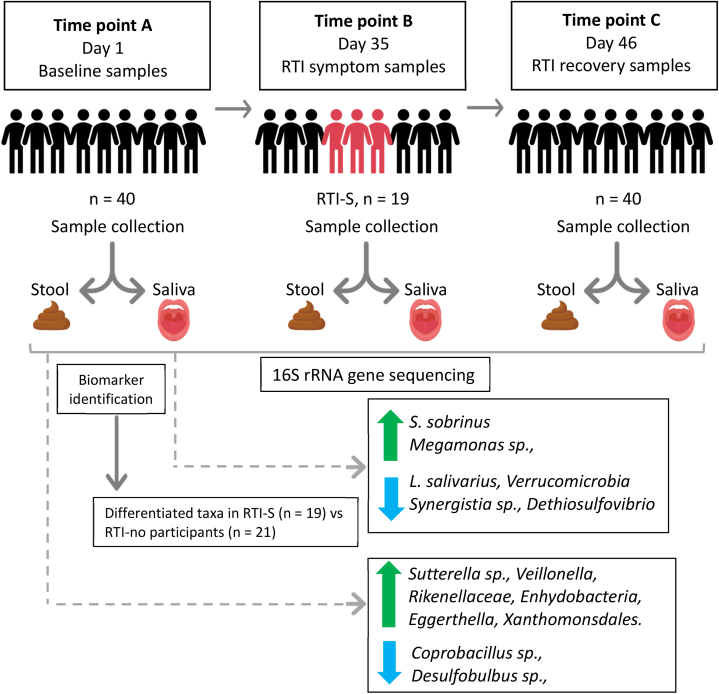
Study design and microbial biomarkers. Each participant provided saliva and stool baseline samples and end of study samples. During the study if they experienced RTI symptoms they also provided additional samples. We used the OMNIgene® ORAL and OMNIgene® GUT kits for the collection of saliva and stool, respectively. A total of 40 participants provided samples and 19 acquired RTI symptoms (RTI-S) whereas 21 remained healthy (no-RTI). Baseline samples comparing RTI-S and no-RTI participants were analysed using 16S rRNA gene sequencing for amplicon sequence variants (ASVs) and biomarker tests (LEfSe and random forest MDA classifier) revealed enhanced microbes in the oral cavity were *S. sobrinus* and *Megamonas* sp., and depletion of *Lactobacillus salivarius*, Synergistetes, Verrucomicrobia and Dethiosulfovibrio. Enhanced gut microbes were *Sutterella* sp., *Veillonella*, Rikenellaceae, *Enhydobacteria*, *Eggerthella, Xanthomonsdales* and depleted were *Desulfobulbus* and *Coprobacillus*.

This study was designed to collect longitudinal participant stool (gut) and saliva (oral) samples at specific time points including self-reported socio-demographic data during the study duration. We hypothesised some participants may develop respiratory symptoms and some would remain healthy throughout the study. Our secondary objective as part of the study design was to determine microbial biomarkers of susceptibility to respiratory infection from baseline oral-gut samples collected at the start of the study comparing RTI-S to no-RTI participants. We present a recruitment diagram (Fig. 1) and graphical study design diagram, further described in the results section (Fig. 2).

### 2.3 Participant recruitment

Primary care practices were invited to express interest in taking part in the study via the National Institute for Health Research (NIHR) Clinical Research Network (CRN). Practices were asked to send out study invitations to healthy patients aged between 18 and 70 years and living independently, using diagnostic codes in the medical records to exclude patients that present conditions which are considered confounders for the gut and oral microbiota, such as: a condition affecting the immune system including but not limited to for example, lupus; without capacity to consent; with a severe life-limiting illness; and those with conditions known to predispose to infection (chronic lung disease, cystic fibrosis, and type 1 and 2 diabetes and who are at greater risk of developing community-acquired RTIs including, but not limited to, chronic obstructive pulmonary disease [COPD], asthma, and bronchiectasis). The practices mailed eligible patients an invitation letter, consent form, and participant information sheet. Those who were willing to participate were asked to return their signed consent form to the study team using prepaid envelopes. The study ran between November 2019 and May 2020. Participant recruitment took place between November 2019 and January 2020. Study recruitment was closed prematurely in February 2020 due to the COVID-19 pandemic in the UK.

### 2.4 Metadata collection

Upon receipt of the participant signed consent form, the study team emailed or telephoned the patient to confirm eligibility and provide instructions regarding baseline data completion online, after which point the participant was fully enrolled. Participants were requested to complete a questionnaire for the collection of covariates used as metadata for microbiota analysis, example questions were: age, sex, height, weight, RTI symptoms, ethnicity, education, employment, public transport usage, history of RTIs in previous 12 months, handwashing, smoking, alcohol, dietary choice, probiotics, supplements, other medication, pet ownership, RTI symptoms, GP visits (a full list of all questions can be seen in supplementary Table S1).

Self-reported RTI symptoms were entered into the database in response to weekly e-mails requesting participants to respond with a ‘yes’ or ‘no’ to the query asking if they had developed at least one or more ‘new’ RTI symptom at any time during the study. Symptoms included: a blocked and/or runny nose, earache and/or ear discharge, sore throat, cough, chest symptoms including breathing faster than normal, wheeze and/or whistling chest (also shown in supplementary Table S1). A positive response prompted the participant to complete a short symptom resolution questionnaire, which included information on what symptoms they had and when they started, whether they consulted with their primary care clinician for their symptoms, took any medication for their symptoms, and took time off work due to their symptoms. From this point, weekly emails commenced asking participants whether their symptoms had fully resolved. Once symptoms had fully resolved the participant was asked to provide post-RTI sample and stool samples and their active study period ended. A negative response resulted in continued weekly emails, asking whether they had developed new RTI symptoms for the duration of the study, or until they responded ‘yes’. For participants who did not report any RTI symptoms and remained healthy during the study, they continued to receive weekly emails until the end of the study period.

At the end of the study, all participants including those who reported RTI symptoms (RTI-S) and those that did not (no-RTI) were prompted to complete a brief end of study questionnaire. This was a condensed version of the symptom resolution questionnaire which simply asked the participant for feedback on the study. Once completed, the participant active study period ended.

### 2.5 Stool and saliva collection

Stool and saliva samples were self-collected by each participant at three time points:

- *Time point A (baseline samples):* Collected within 3 days of recruitment to the study if the participant was RTI symptom-free. If the participant had RTI symptoms at recruitment, they were asked to collect their samples once they had been free of symptoms for at least 2 days.
- *Time point B (RTI symptomatic samples [RTI-S]):* Collected at onset of RTI symptoms or within 2 days of RTI symptoms being reported and reminders were emailed to participants to collect their samples if they reported having RTI symptoms.
- *Time point C (RTI recovery or end of study samples):* Collected once RTI symptoms had resolved fully for at least 2 consecutive days from RTI-S participants. For participants who remained healthy (no-RTI), this was an end of study sample.

Stool and saliva samples were collected into OMNIgene®GUT and OMNIgene®ORAL (DNAGenotek) collection tubes, respectively. These kits contain a stabilisation reagent which enables storage of samples at room temperature for 60 days prior to DNA extraction and are highly convenient for sample collection at-home. Participants were provided with detailed sample collection instructions, including a link to the manufacturer’s video instructions. Collected specimens were posted by post directly to the research laboratory for nucleic acid extraction and subsequent sequencing for 16S rRNA microbiota analysis.

### 2.6 Nucleic acid extraction

Bacterial genomic DNA (gDNA) and RNA was extracted using QIAamp One-For-All kit (QIAGEN) according to the manufacturer’s instructions and quantified using a Qubit™ fluorometer. Aliquots of nucleic acids were transported on ice to the clinical diagnostics laboratory for reverse-transcriptase-PCR (RT-PCR) analysis.

### 2.7 Microbiota profiling

The V4 region of the bacterial 16S rRNA gene was amplified using V4 primers 515F (5′ – GTGCCAGCMGCCGCGGTAA – 3′) and 806R (5′ – GGACTACHVGGGTWTCTAAT - 3′) [21]. A library of 16S amplicons were barcoded, sequenced on Illumina NovaSeq 6000, then trimmed of barcodes by Novogene™ services. All raw sequence reads (FASTA files) were processed in-house using pipelines and analysis listed in the following section.

### 2.8 Bioinformatics and statistical analysis

The sequence reads were processed using Quantitative Insights into Microbial Ecology tool (QIIME2-2020.2) [22]. Raw sequences were denoised and chimeras removed with DADA2 [23]. To improve the accuracy of phylogenetic placements, amplicon sequence variants (ASVs) were aligned with MAFFT [24] (via q2-alignment) and constructed using the fasttree2 [25]. Taxonomic classification was preformed using the q2-feature-classifer [26] classify‐sklearn naïve Bayes taxonomy classifier and Greengenes 13_8 (99%) reference sequences [27]. The feature table, taxonomy, phylogenetic tree and sample metadata were then combined into a Phyloseq object using QIIME2R [28] via qza_to_phyloseq.

All further analysis was done in R (v3.6.0) [29] via RStudio (v1.3.1073) using microbial scripts. Figures were produced using the package ggplot2 [30]. Phyloseq (v1.29.077) [31] was used following a published workflow [32]. Potential sample contaminants were identified by prevalence in negative control samples and removed using the R package ‘decontam’ [33]. Sample processing, and bioinformatic analyses were tested using samples spiked with *Bordetella* sp. prior to DNA extraction. ASVs with Phylum classifications of ‘NA’ or ‘uncharacterised’ and any Phyla with a total of fewer than five ASVs whose provenance suggested low level contamination were removed using the subset_taxa() function. Less than 5% of the total number of ASVs were removed from either the saliva or stool sequences prior to diversity and biomarker analysis. Alpha diversity was performed using the estimate_richness() and stat_compare_means() was used to compare groups using the nonparametric Wilcoxon test. Beta diversity was determined by plotbeta for hierarchical clustering by Bray–Curtis dissimilarity using Principal component analysis (PCoA). Betatest() function was used to calculate the permutation multivariate analysis of variance (PERMANOVA) values for variables of interest. Core microbial analysis was performed using the ‘microbiome’ vignette using heatmaps for visualisation [34].

### 2.9 Microbial biomarker analysis

Differentially abundant ASVs between groups were identified by linear Discriminant Analysis (LDA) coupled with the effect size (LEfSe) [35] (LDA score >2, *p* < 0.05). The ldamarker() function was used for LEfSe analysis based on Kruskal-Wallis and LDA analysis. The DESeq2 package using an adjusted -value cut-off of 0.05 and a Log2 fold change of 1.5 was also applied to the saliva and stool bacterial sequence data [36]. A random forest classifier was used to rank the importance of the mean decrease in accuracy (MDA) for each taxa using the ‘microbial’ package [37]. DESeq2 and LEfSe methods were used to identify taxa that are differentially expressed across the samples, and random forest model was used to rank taxa with largest contribution towards infection classification.

### 2.10 Diagnostics of common respiratory microbes

The sample collection period overlapped with the start of the COVID-19 pandemic in the UK. To determine the presence of coronavirus all samples were tested for the Beta-CoV E gene and SARS-CoV-2 S gene targets. A clinically validated 45-microbe low density TaqMan Array Card (TAC) (Applied Biosystems, Foster City, CA, USA) was used to detect common respiratory microbes, using exogenous controls (T4 and MS2 bacteriophage gene targets), endogenous human controls (human 18S rRNA and RNase-P gene targets), see supplementary Table S2 for a full list of microbe gene targets [38].

Samples were run in 3 batches, amplified, and analysed using a Life Technologies Custom TaqMan Low Density Array system on an Applied Biosystems Life Technologies ViiA-7TM real-time PCR system as described elsewhere [39]. A cycle threshold (Ct) value < 38 for any gene target was reported as a positive result. Incidence of microbe carriage prevalence was calculated as the number of positive results as a percentage of all positive results. The Pearson’s chi-squared test with a Bonferroni-adjusted P value was used to determine significance between observed proportions of microbial carriage.

## 3 Results

### 3.1 Study population characteristics

The flow-chart (Fig. 1) describes participant recruitment and retention including those that reported RTI symptoms (RTI-S) and those who remained healthy (no-RTI). A total of 12 general practice (GP) surgeries expressed an interest in joining the study and eventually 3 agreed to participate, although one was excluded due to pandemic disruptions. Two GP practices, both within central Bristol, England, were recruited to the study and serve a broad range of socio-economic populations. We invited 3000 patients from these surgeries and the recruitment rate was 1.33%, see Fig. 1, (inclusion and exclusion criteria are described in the Methods section). A total of 16 participants either did not collect and return time point B (RTI symptom samples) or end of study samples. It is possible samples were lost in transit due to disruption to postal services during the first UK COVID-19 lockdown. Also, it was not possible to determine whether 9 participants were RTI-S or no-RTI status, so their data were removed from the study. Of the remaining 19 RTI-S participants a further 2 sample sets at time point C, the end of the study samples, which were also lost in transit and were not returned. In total 40 paired stool and saliva baseline samples were analysed for microbial biomarkers as shown in the study design diagram (Fig. 2).

We intended to collect health-related risk factors from participant medical notes, including current medications and antibiotic prescriptions in the 12 months prior to recruitment, however, due to the pandemic we were unable to access these data. Descriptive statistics for the most relevant covariates from 40 participants are shown in Table 1. For no-RTI participants (*n* = 28) 50% were female and their mean (+ standard deviation [SD]) age was 48.3 (+2.8) years. For this group, a total of 42 stool and saliva samples were collected at time points A, baseline, and C, end of study. For the RTI-S participants (*n* = 19), 74% were female and the mean age 46.1 (+3) years. Stool and saliva samples were collected at time point A, baseline, s (n = 38), B, RTI-symptom samples (n = 38) and C, RTI-recovery samples (n = 34). There was a mean of 34.8 (+7.5) days from the study start until participants reported an RTI symptom. There was a mean of 14 (+2.5) days between collection of RTI-symptom and RTI-recovery samples. The main RTI symptom was characterised as a sore throat in 68.5% of cases, followed by a runny nose in 63.2% of cases.

**Table 1:** Participant characteristics.

Descriptive characteristics	Participants	
	RTI-S	No-RTI
Number of participants (*n* = 40)	19	21
Gender (%)		
Females	74	50
Males	26	50
Age, years (mean + SD)	46.1 (3)	48.3 (2.8)
BMI (mean + SD)	24.1 (0.9)*	24.9 (0.8)
Ethnicity (%)		
White	100	95
Asian	0	5
Education (%)		
Higher education	95	57
Further education	5	43
Is there a smoker at home? Yes (%)	0	9.5
Do you have a pet? Yes (%)	50*	38
Most common pet..	Dog/s	Cat/s
RTI episodes in previous 12 months (mean + SD)	1.7 (0.3)*	1.8 (0.3)
**Samples collected at time point (TP):**		
TP-A: Baseline (stool & saliva)	19 & 19	21 & 21
TP-B: RTI symptoms (stool & saliva)	19 & 19	0
TP-C: Study end (stool & saliva)	17 & 17*	21 & 21
Total samples	110	84
Days from TP-A to TP-B (mean + SE)	34.8 (7.5)	NA
Days from TP-B to TP-C (mean + SE)	46.1 (7.7)	NA
Days from TP-A to TP-C (mean + SE)	80.9 (7.2)	63.7 (2.9)
**RTI symptoms at TP-B:**		
Length of episode, days (mean + SE)	14 (2.5)	0
Severity Likert scale:1 = mild to 10 = severe (mean + SE)	5.1 (0.5)	0
Runny nose: Yes (%)	63.2	NA
Cough: Yes (%)	36.8	NA
Sore throat: Yes (%)	68.5	NA
Chest symptoms: Yes (%)	5.3	NA

Self-reported clinical symptoms, socio-demographic data and biological samples (stool and saliva) were collected from participants with RTI symptoms (RTI-S) and those who remained healthy (no-RTI). Abbreviations: Not applicable (NA). *Missing data: two participants didn’t complete the questionnaire data and their ‘end of study’ time point C samples weren’t received in the laboratory.

### 3.2 Carriage of respiratory pathogens

To determine carriage of common respiratory tract pathogens in participants, all saliva samples and RTI-S stool samples were analysed by RT-PCR using a 42 pathogen Taqman Array Card (TAC). Pathogen carriage was defined as the number of positive results for a specific pathogen as a percentage out of all results (Table 2).

**Table 2:** Oral carriage of respiratory pathogens from participants with RTI symptoms (RTI-S) and those who remained healthy (no-RTI) at three time points (TP).

	Carriage prevalence percent (%)				
Respiratory pathogen	RTI-S			No-RTI	
	TP-A (*n* = 19)	TP-B (*n* = 19)	TP-C (*n* = 17)	TP-A (*n* = 21)	TP-C (*n* = 21)
CoNS	73.7*	84.2	76.5	42.8*	47.6
*S. aureus*	0	5.3	5.9	14.2	9.5
*F. necrophorum*	5.3	5.3	0.00	4.8	4.8
*H. influenzae*	26.3	31.6	29.4	19.0	14.2
*M. pneumoniae*	5.3	5.3	0.0	9.5	0.0
*S. pneumoniae*	26.3	10.5	23.5	23.8	9.5
*M. catarrhalis*	10.5	5.3	17.6	14.2	9.5
Parechovirus	0	5.3	0.00	0	0
Rhinovirus	0	5.3	5.9	0	0
Rhinovirus-2	5.3	5.3	5.9	0	0
Influenza A	0	15.7	11.8	4.7	9.5

*Bonferroni-adjusted, *p* = 0.0045 (Pearson’s chi-squared, *p* = 0.049). All RTI-S and no-RTI saliva and RTI-S stool samples tested negative for human coronavirus Beta-Cov, E gene and SARS-CoV-2, S gene. Abbreviations: Coagulase-negative staphylococci (CoNS), time point (TP): A, baseline; B, RTI symptoms; C, end of study/RTI-recovery.

Coronavirus SARS-Cov2 was not detected in any sample. Influenza (subtype A) carriage in RTI-S participants at baseline, RTI-symptom and RTI-recovery samples was zero, 15.7% and 11.8% respectively. For no-RTI participant it was 4.7% at baseline and 9.5% at the end of study. At all the time points, for both RTI-S and no-RTI participants, there was carriage of *H. influenzae*, *S. pneumoniae, M. catarrhali*s, including high carriage in RTI-S, time point B samples (RTI-symptom samples). Taken together these observations suggest it wasn’t possible to determine a definitive cause of the RTI from these analyses.

In RTI-S participants coagulase-negative *Staphylococcus* (CoNS) carriage at baseline (A), RTI-symptoms (B) and RTI-recovery (C) samples was 73.7%, 84.2% and 76.5%, respectively, whereas in no-RTI participants it was 42.8% at baseline (A) and 47.6% at end of study samples (C). For baseline samples (A) the difference between CoNS carriage of 73.7% and 42.8% was not statistically significant ([Pearson’s chi-squared, *p* = 0.049] Bonferroni-adjusted, *p* = 0.0045). In no-RTI participants *S. aureus* carriage at baseline (A) and end of study (C) samples ranged from 9.5 to 14.2% compared to RTI-S participants which showed no carriage at baseline (A), RTI-symptoms (B) at 5.3% and RTI-recovery (C) at 5.9%.

### 3.3 Oral and gut microbiota

Oral and gut microbiota (OM and GM) of participants were defined by 16S rRNA V4 region profiling where the OM yielded a median of 104,201 reads per sample from which 7692 ASVs were taxonomically assigned, and the GM samples produced a median of 102,627 reads from which 8009 ASVs were taxonomically assigned. For both microbiota the relative proportions of phyla, genera, and species at baseline (A) comparing RTI-S to no-RTI participants can be seen in supplementary Figure S1 (oral [A] and gut [B]).

#### 3.3.1 Core microbiota

**Fig. 3:**
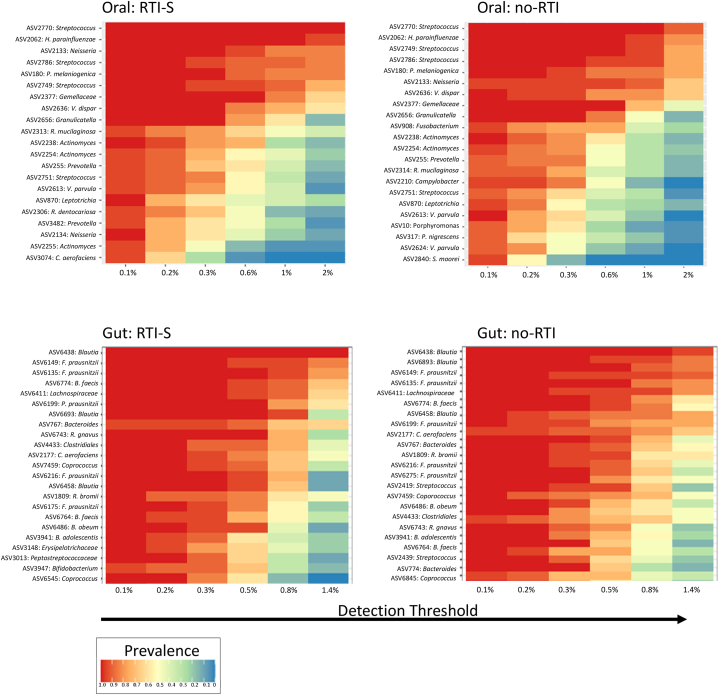
A heatmap representing core microbes in oral (top) and gut (bottom) baseline collected samples from participants who acquired RTI symptoms (RTI-S) compared to those who remained healthy (no-RTI). The x axis shows the percentage detection threshold of amplicon sequence variants (ASV) and the y axis displays ASV prevalence.

The core taxa of OM and GM (taxa present in 90% of each body niche samples at a relative abundance of at least 1%) were identified (Fig. 3). Out of a total of 2121 taxa (tt) the top three core oral ASVs in RTI-S participants were *Streptococcus* (ASV 2770), *H. parainfluenzae* (ASV 2062) and *Neisseria* (ASV 2133) and in no-RTI participants (2846 tt) this was *Streptococcus* (ASV 2770 and 2749) and *H. parainfluenzae* (ASV 2062). The core gut ASVs in RTI-S participants (3161 tt) were *Blautia* (ASV 6439) and *F. prausnitzii* (ASVs 6135 and 6149) and in the no-RTI participants (3258 tt) were *Blautia* (ASVs 6439 and 6883) and *F. prausnitzii* (ASV 6149).

#### 3.3.2 Alpha and beta diversity

**Fig. 4:**
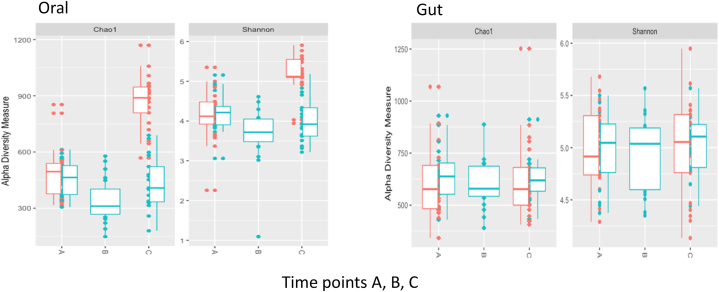
Oral and gut microbial richness in participants who acquired RTI symptoms (blue) compared to those who remained healthy (coral) at all study time points. The x axis shows the time when samples were collected; A, baseline; B, RTI symptoms; C end of study/recovery from RTI. The y axis shows alpha diversity measure value as assessed by Chao1 and Shannon.

Alpha diversity values were measured using the Shannon Diversity Index and Chao1 and significance values for the OM and GM are shown in Table 3 and Fig. 4. In baseline microbiota there was no significant difference in diversity between RTI-S and no-RTI participants. In the OM at end of study/RTI-recovery samples (C) there was a significant difference in diversity between the no-RTI and RTI-S participants and when comparing baseline (A) and end of study (C) for only the no-RTI participants. In the GM diversity shifts were not observed, they remained stable throughout the study.

**Table 3:** Oral and gut microbial diversity at the start and end of the study.

Sample covariates	Oral		Gut	
	Chao1	Shannon	Chao1	Shannon
**TP-A samples:**				
No-RTI (21) vs RTI-S (19)	0.80	0.82	0.48	0.82
CoNS: -ve (n = 17) vs +ve (n = 23)	0.30	0.48	na	na
**TP-C samples:**				
No-RTI (21) vs RTI-S (17)	5.5 × 10^−7^	6.6 × 10^−6^	0.76	0.40
CoNS: -ve (n = 15) vs +ve (n = 23)	6.8 × 10^−3^	0.10	na	na

Microbial richness was assessed using the Shannon diversity index and Chao1 measures, *p* values. Abbreviations: not applicable (na), RTI symptoms (RTI-S) and no RTI symptoms (no-RTI), coagulase-negative *Staphylococci* (CoNS), time point (TP): A, baseline; C, end of study/RTI-recovery.

Saliva samples which tested positive for CoNS (*n* = 62) demonstrated a significant reduction in diversity compared to those that tested negative for CoNS (*n* = 35) (*p* = 2.5 × 10^−3^). Other factors affecting the GM diversity was the month of sample collection and pet ownership (covariates of interest with significance values can be seen in supplementary Table S3).

Beta diversity, Bray-Curtis PCoA plots revealed no visually apparent clusters in any microbiota at any time points (supplementary Figure S2). However, when all samples at any time point were tested using PERMANOVA the OM indicated significant differences between RTI-S vs no-RTI participants (*p* = 0.001), CoNS positive vs CoNS negative (*p* = 0.002) whereas for the GM there were no significant differences between no-RTI vs RTI-S participants (*p* = 0.139).

#### 3.3.3 Microbiota biomarkers

**Fig. 5:**
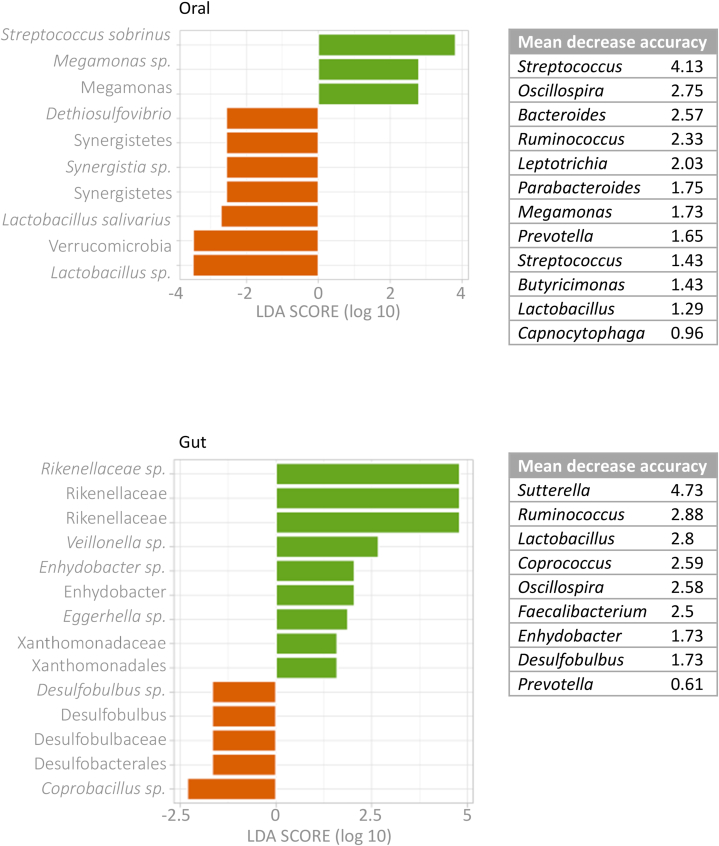
Microbial biomarkers in oral (top) and gut (bottom) baseline samples from participants who acquired RTI symptoms compared to those who remained healthy. Linear discriminant analysis effect size (LEfse) log 10 scores were used to identify RTI-S (green) and no-RTI (orange) biomarkers in oral and gut baseline samples. Tables on the right-side show top ranked taxa identified from a random forest model as mean decreased accuracy.

Samples collected at the start of the study, the baseline samples (A), from those who subsequently acquired RTI symptoms (RTI-S) were compared to those that remained healthy (no-RTI) by analyses to identify potential microbial biomarkers of susceptibility to RTI. We performed two biomarker tests, LEfSe based on the Kruskal-Wallis test and LDA (log_10_), Random Forest model (MDA) (Fig. 5). Biomarker for the end of the study/RTI-recovery (C) samples can be seen in supplementary Figure S3 (oral [A] and gut [B]).

The OM of RTI-S participants compared to no-RTI participants by LEfSe analysis revealed an increased abundance of *Streptococcus sobrinus* and *Megamonas* but decreased abundance of Synergistetes, Verrucomicrobia, Dethiosulfovibrio and *Lactobacillus salivarius* whereas the Random Forest MDA identified *Streptococcus* (4.13) as the top-ranking biomarker.

The GM of RTI-S participants compared to no-RTI participants by LEfSe analysis revealed an increased abundance of Rikenellaceae, *Veillonella*, *Enhydobacteria*, *Eggerthella* and *Xanthomonsdales* and decreased abundance of *Desulfobulbus* and *Coprobacillus* whereas the Random Forest MDA identified *Sutterella* (4.73) as the top-ranking biomarker.

## 4 Discussion

Understanding the role of the oral-gut microbiota in acquisition of an RTI is an area of intense interest. In our feasibility study the primary objective was to determine whether an urban adult cohort could be recruited from general practitioners in the Bristol region would participate in this longitudinal study and collect paired saliva and stool samples for subsequent microbial analysis. Although we found low recruitment rates, the retention rate was high and based on the number of samples collected we investigated oral-gut microbial biomarkers for community acquired RTI. We hypothesised that oral-gut microbes from participants samples prior to RTI acquisition compared to those who acquired a RTI may have differences in their microbial profiles and here we found differential abundances for specific taxa. Although it is unknown if these microbial biomarkers went on to influence pathogen acquisition or pathobiont proliferation, our study shows promise for identification of oral-gut biomarkers for early diagnosis of respiratory infection susceptibility.

Numerous studies have demonstrated that gut microbiota disruption occurs during and following respiratory infection by viral and or bacterial pathogens [40] but studies which describe predicted acquisition of an RTI using baseline multi-microbiota are generally lacking. One study identified specific gut microbiota markers in baseline samples were associated with an improved immune response and reduced adverse events following COVID-19 vaccines [14] while another sampled oral-gut microbiota by collecting throat and stool samples respectively, following 53 patients infected with SARS-CoV-2 through to recovery to reveal associations between microbial species with disease severity and viral load [41].

In our study, the core oral microbiota comprised commonly found *Streptococcus* and *H. parainfluenzae*. The latter is one of the most abundant and prevalent species in the oral cavity and dental plaque of healthy individuals, yet, it is a member of the HACEK (*Haemophilus*, *Aggregatibacter* [previously *Actinobacillus*], *Cardiobacterium*, *Eikenella*, *Kingella*) group that is associated with diseases, including endocarditis and lung cancer [42,43]. Core microbial signatures can be useful for predicting oral health and possibly RTI [44]. In the oral microbiota two ‘stomatotypes’ exist, with stomatotype-1 characterised by abundant *Neisseria* and *Haemophilus* while stomatotype-2 is associated with a high level of *Prevotella* and *Veillonella* [45]. At the start of the study in healthy participants the core microbiota is indicative of stomatotype-1 but shifts to stomatotype-2 at the end of the study including an increase in pathobiont *P. melaninogenica* (supplementary information). An increased relative abundance of *P. melaninogenica* in the nasopharynx of children has been associated with severe influenza [46]. It’s not clear why oral microbes of healthy participants undergoes a stomatotype shift, but the month of sample collection was associated with a change in the alpha diversity of samples and thus it is possible that this is associated with this change in the core microbiota (the start and end of study sample collection was October and March respectively).

Comparing the baseline oral microbiota of participants who acquired an RTI to those who did not revealed acquisition was associated with enhanced relative abundance of *S. sobrinus* and *Megamonas*. *S. sobrinus* is a pathobiont that is strongly linked to the development of dental caries and oral disease [47]. Interestingly, it revealed depletion of beneficial bacteria common in saliva, *L. salivarius,* which has known probiotic, antimicrobial, and immunomodulatory properties [48]. Strikingly, the incidence of carriage of CoNS was greater in participants that acquired an RTI compared to those who remained healthy. CoNS are opportunistic pathogens that colonize the skin and mucous membranes of the oral cavity in healthy individuals [49]. CoNS have been implicated as a significant etiological factor of RTIs including laryngological infections and viral infections such as chronic rhinosinusitis which is a viral inflammation of the mucous membranes of the rhino-sinus and pharyngitis, yet disease mechanisms remain largely unknown [[50], [51], [52], [53]]. Although, there was no evidence to suggest CoNS caused the RTIs in participants, we found a possible association between CoNS and a less diverse microbiota. The presence of CoNS as a potential biomarker of RTI susceptibility deserves further investigation.

The gut microbiota was substantially more diverse than the oral cavity, as expected but the core microbiota of RTI-S and no-RTI participants was largely similar, with high levels of the genus *Blautia* and species *Faecalibacterium prausnitzii*. These bacteria are usually considered beneficial gut bacteria and have a close relationship, but a recent review has implied that their influence on health and disease is dependent on the species of *Blautia* present [54]. Comparison of baseline samples of RTI-S to those of no-RTI participants identified a higher relative abundance of *Veillonella* and *Sutterella*. Relatively high levels of *Veillonella* was observed in the gut microbiota of COVID-19 patients compared to healthy controls and is a key microbe in colorectal associated-cancer and a putative gut biomarker for cystic fibrosis [55,56]. The clinical significance of *Sutterella* is unknown but has been associated with enrichment in patients with intestinal disease [57,58]. Although our study overlapped the beginning of the COVID-19 pandemic in the UK, participants did not report any confirmed cases of COVID-19, we did not detect SARS-CoV2 in any sample and the GM of RTI-S participants showed a depletion of *Coprobacillus* whereas this genus was enriched in patients with COVID-19 [59]. Our results suggest that the oral microbiota shows more marked differences than the GM associated with acquisition of an RTI but a multi-microbiota approach that simultaneously test two or more host sites provides an holistic approach to understanding subtle microbial influences on susceptibility to respiratory infection.

## 5 Study limitations

While we conclude that we have demonstrated the feasibility of this approach, our study has several limitations. Firstly, as a feasibility study the primary aim was to demonstrate whether our study design was suitable for the collection of biological samples, self-reported socio-demographic and clinical data could be collected, with the ultimate view of informing the design of a larger cohort study. While we acknowledge the small sample size and the fact it’s not possible to accurately predict respiratory infection in the community with the information presented in this research paper, longitudinal microbial studies are extremely valuable for understanding microbial temporal patterns to predict respiratory infection in the community. The UK national lockdown in response to the COVID-19 pandemic in March 2020 caused the premature closure of this study, which forced us to stop recruitment from February 2020 onwards. We also experienced postal disruption which resulted in several samples lost in transit. Furthermore, due to the UK-wide restrictions on primary care data during the pandemic we were not able to collect health-related risk factors from medical notes. This study relied on self-reporting of RTI symptoms and severity, so access to medical history and current health records would have strengthened designation of RTI severity, identified prescribed medications, and enabled diagnostic confirmation of causative pathogen. Currently, we are unable to identify the likely cause of the RTIs recorded in our study, and we recognise that different factors may determine the acquisition of different pathogens, and this could affect our analyses where we have grouped all RTIs into one group. However, this was not possible under lockdown restrictions. While our participant questionnaire was used to assess a broad range of microbiota covariables including dietary and lifestyle (alcohol intake, physical activity) factors, we observed microbiota diversity between the months of sample collection. Seasonal variation of the GM has been well documented and thus this factor must be taken into consideration in further seasonal RTI microbiota studies as a possible confounder [60]. The study was designed to enrol individuals aged between 18 and 70 years during the winter months. To ensure enough self-reported clinical events we chose to sample during a UK winter season when RTIs are prevalent. There was considerable perturbation of oral microbial diversity in samples from participants who remained healthy throughout the study whereas the oral microbiota of participants who acquired a RTI returned to a similar level of compositional diversity. It’s not clear why this was the case, but we might assume this was due to collection of samples on different months, as noted with seasonal confounder effects. We considered network microbiota analysis, however due to low participant numbers we predict this approach will lack statistical power when comparing network clusters across niches. We used RT-PCR to assess pathogen carriage as a microbiota covariable however microbe load was not considered as a covariate in this analysis. In our study we choose biomarker methods, LEfSe and a Random Forest classifier using MDA. They were applied for a robust approach alongside identification of the core microbiota and RT-PCR to identify common respiratory pathobionts and pathogens. No pipeline is considered standard or universal for such analyses and importantly further work to supplement biomarker findings such as the identification of host immunological molecules and or microbial products should be considered. We targeted patients registered at local GP practices in the Bristol city urban primary care community setting [61]. Although this was a suitable primary care setting, we acknowledge further studies should be conducted in rural areas and different urban primary care settings for comparisons. Finally, we found individual response rates were low, even though we compensated participants with a shopping voucher, but once participants were recruited into the study retention rates were high as seen in other RTI feasibility studies [62].

In summary, this feasibility study has shown that it is possible to recruit participants from a primary care community setting, for the collection of stool and saliva samples and self-report RTI symptoms for the duration of the study of more than 80 days. This study contributes to the emerging picture that microbial biomarkers of susceptibility to RTI are identifiable suggesting predisposition to the increased health risk of respiratory infection. In the future they could predict vulnerable members of the community at risk of respiratory infection and minimise the use of unnecessary health care associated treatments.

## Author contribution statement

Claire A Woodall: Conceived and designed the experiments; Performed bioinformatic tests and experiments; Analysed and interpreted data; Wrote the paper.

Ashley Hammond: Conceived and designed the experiments; Analysed and interpreted the data.

David Cleary; Andrew Preston; Ben Pascoe; Samuel K Sheppard: Analysed and interpreted the data.

Peter Muir: Contributed reagents, materials, analysis tools or data.

Alastair D Hay: Conceived and designed the experiments; Analysed and interpreted the data.

## Data availability statement

Data will be made available on request.

## Additional information

Supplementary content related to this article has been published online at: https://www.cell.com/heliyon/home.

For Open Access, the author has applied a CC BY public copyright licence to any Author Accepted Manuscript version arising from this submission.

## Declaration of competing interest

The authors declare that they have no known competing financial interests or personal relationships that could have appeared to influence the work reported in this paper.
