# Supplementary material for: Oral and gut microbial biomarkers of susceptibility to respiratory tract infection in adults: A feasibility study
Source: Heliyon. 2023 Jul 28;9(8):e18610. doi: 10.1016/j.heliyon.2023.e18610 (PMC10432180; doi:10.1016/j.heliyon.2023.e18610)
Supplement: Multimedia component 1 [file mmc1.docx]

| General questions | Participant response |
| --- | --- |
| General practitioner surgery name | Text* |
| Recruitment start | Date |
| How did you hear about this study? | Text |
| Stool sample collection   - Time point A (start of study/baseline) - Time point B (during RTI) - Time point C (end of study/RTI recovery) | Date  Date  Date |
| Saliva sample collection   - Time point A (start of study/baseline) - Time point B (during RTI) - Time point C (end of study/RTI recovery) | Date  Date  Date |
| Age | Number |
| Sex | Text |
| Ethnicity | Text |
| Education | Text |
| Employment | Text |
| Job title | Text |
| In your job do you encounter children? | Yes/No |
| Do you use public transport?   - State public transport frequency? | Yes/No Number |
| Your weight in kilo grams | Number |
| Your height in metres | Number |
| Your body mass index | Number |
| How often do you wash your hands? | Number |
| How often do you exercise? | Number |
| Have you ever smoked?   - How often do you smoke | Yes/No Number |
| How often do you drink alcohol per week? | Number |
| How often do you eat meat? | Number |
| How often do you eat chicken? | Number |
| How often do you eat red meat? | Number |
| How often do you eat processed meat? | Number |
| How would you describe your diet choice? | Text |
| Do you have a dietary medical issue? | Text |
| How often do you eat fruit? | Number |
| How often do you eat vegetables? | Number |
| Do you take probiotic supplements?   - Name and frequency of supplements | Yes/No Text/number |
| Do you take herbal homeopathic remedies?   - Name and frequency of remedies | Yes/No Text/number |
| Do you take over the counter medications   - Name and frequency of medications | Yes/No Text/number |
| How many adults are in the home?   - What are the ages of the adults in the home? | Number  Number |
| How many children are at home?   - What are the age of the children at home | Number  Number |
| Do your children attend day care? | Yes/No |
| Do your children attend school? | Yes/No |
| How many bedrooms does your home have? | Number |
| Do you live with someone who smokes at home? | Yes/No |
| Do you have any pets at home?   - What type of pet do you have? | Yes/No  Text |
| How would you like the study team to communicate with you? | Text |
| How many RTIs have you had in the previous 12 months? | Number |
|  |  |
| Self-reported RTI symptoms: |  |
| Have you developed at least one or more ‘new’ RTI symptom at any time during the study? | Yes/No  – if yes then: |
| When did your RTI symptoms start? | Date |
| Are you experiencing a runny and or blocked nose? | Yes/No |
| Are you experiencing a cough? | Yes/No |
| Are you experiencing a sore throat? | Yes/No |
| Are you experiencing any chesty symptoms, a wheeze and/or a whistling chest? | Yes/No |
| Are you breathing faster than normal? | Yes/No |
| Have you had a change in appetite? | Yes/No |
| Describe how unwell you have felt during this RTI?  On a 10-point scale, where 10 is severe and 0 is none. | Number |
| Did you have to visit your GP doctor during your RTI episode? | Yes/No |
| Have you been prescribed antibiotics for this RTI episode? | Yes/No |
| When did your RTI symptoms end? | Date |

* A free text box was provided for participant responses.

**Table S1. Participant questionnaire including self-reported respiratory tract infection (RTI) symptoms.**

| Group | Microbe gene target |
| --- | --- |
| Human controls | 18s rRNA gene |
|  | Rnase P gene |
| Internal controls | Bacteriophage MS2 |
|  | Bacteriophage T4 |
| Viruses | Human adenovirus-C, type 2 |
|  | Human adenovirus B |
|  | Human bocavirus |
|  | Enterovirus |
|  | Enterovirus D68 |
|  | Influenza A CDC DC |
|  | Influenza A H1 2009 ABI #1 |
|  | Influenza A H3 seasonal Cfl |
|  | Influenza Quad AM2 |
|  | Influenza B Bruges |
|  | Influenza B Quad |
|  | Human coronavirus GP2 OC43/HKU1 |
|  | Human coronavirus Beta-CoV E gene |
|  | Human coronavirus Beta-CoV S gene |
|  | Influenza A H1N1/09, R |
|  | Influenza A H1N1/09, S |
|  | Human metapneumovirus |
|  | Human parainfluenza, type 1 and 2 |
|  | Human parainfluenza, type 1 |
|  | Human parainfluenza, type 2 |
|  | Human parainfluenza, type 2 and 3 |
|  | Human parainfluenza, type 3 |
|  | Human parainfluenza, type 4 |
|  | Human coronavirus NL63 |
|  | Human coronavirus OC43 |
|  | Human coronavirus 229E |
|  | Human parechovirus |
|  | Rhinovirus 1 |
|  | Rhinovirus 2 |
|  | Respiratory syncytial virus A |
|  | Respiratory syncytial virus B |
| Bacteria | *Bordetella pertussis* IS481 |
|  | *Chlamydia pneumoniae* |
|  | Coagulase-negative Staphylococcus species (CoNS) |
|  | *Fusobacterium necrophorum* |
|  | *Haemophilus influenzae* |
|  | *Moraxella catarrhalis* |
|  | *Mycoplasma pneumoniae* |
|  | Methicillin resistance gene (MetR) |
|  | *Neisseria meningitidis* |
|  | *Staphylococcus aureus*, nuc gene |
|  | *Streptococcus pneumoniae* |
|  | *Streptococcus pyogenes* |
| Toxins | *Bordetella pertussis* S1 subunit (S1) |
|  | *S. aureus* Panton-Valentine leukocidin toxin (PVL) |

**Table S2. TaqMan Array Card gene targets.**

*Note:* Methicillin resistance gene (Met) is the known marker of methicillin resistance *S. aureus*, Panton–Valentine leukocidin (PVL) toxin is a marker for *S. aureus* virulence and pertussis toxin S1 subunit for *B. pertussis* virulence.


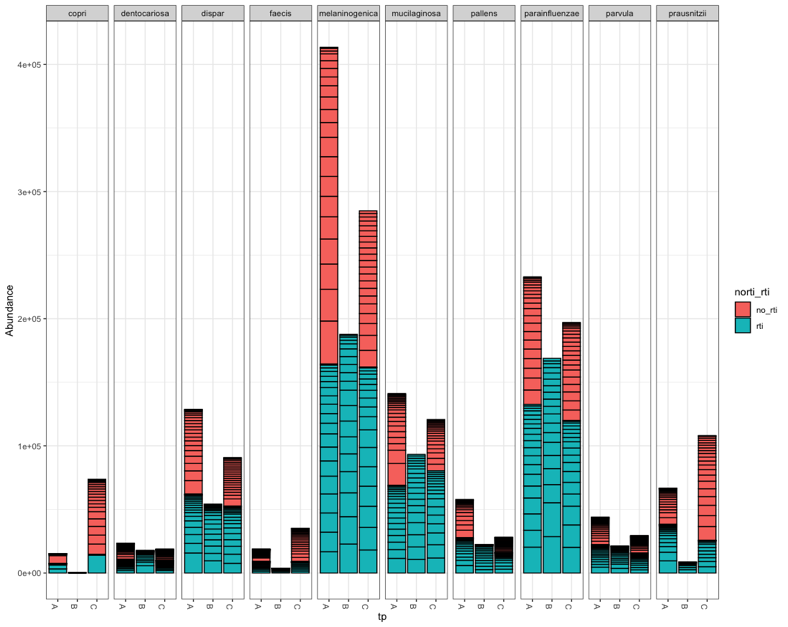

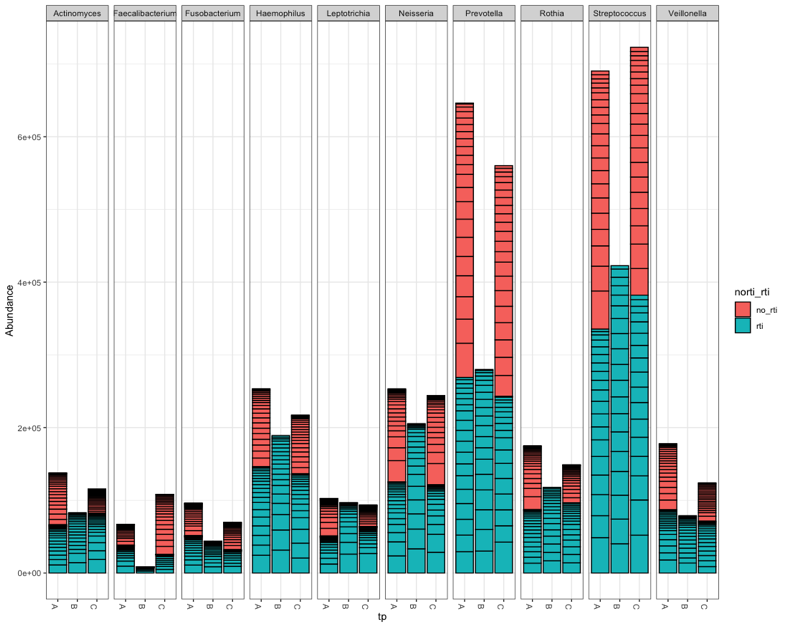


**Figure S1A. Abundant oral microbes in participants who suffered with RTI symptoms (RTI - blue) compared to those who remained healthy (no-RTI – coral) baseline samples, genus (top) and species (bottom).**

The x axis represents sample collection time points, baseline (A), RTI-symptoms (B), RTI-recovery/end of study (C) and y axis the ASV abundance.


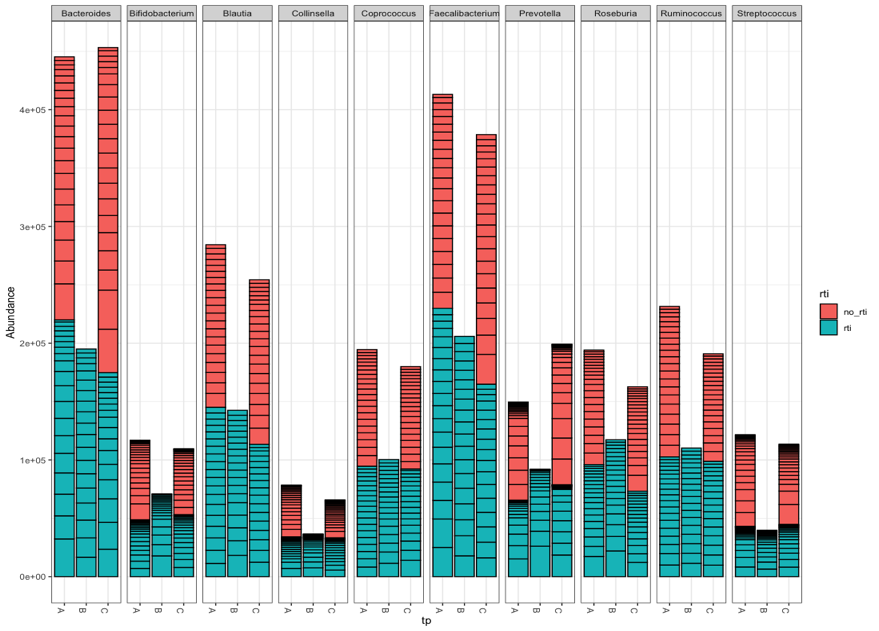

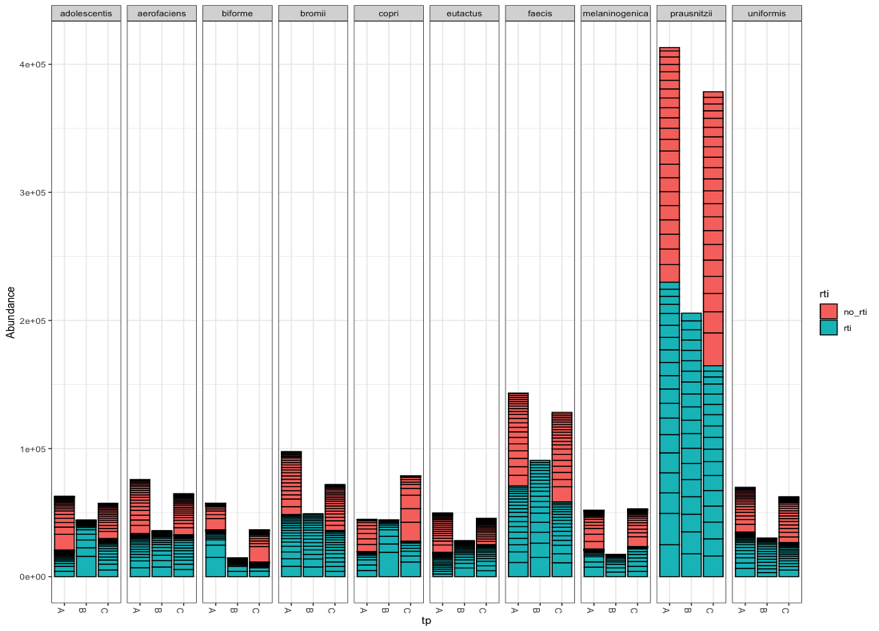


**Figure S1B. Abundant gut microbes in in participants who suffered with RTI symptoms (RTI - blue) compared to those who remained healthy (no-RTI – coral) baseline samples, genus (top) and species (bottom).**

The x axis represents sample collection time points, baseline (A), RTI-symptoms (B), RTI-recovery/end of study (C) and y axis the ASV abundance.

| Covariates of interest | Oral | | Gut | |
| --- | --- | --- | --- | --- |
|  | Chao1, *p* | Shannon, *p* | Chao1, *p* | Shannon, *p* |
| December (n = 15), January (n = 49) | 0.72 | 0.15 | 7 x 10^-4^ | 8 x 10^-6^ |
| January (n = 49), February (n = 29) | 0.93 | 0.75 | 2 x 10^-4^ | 5.4 x 10^-5^ |
| Employed (n = 72), retired (n = 20)* | 0.71 | 0.59 | 0.03 | 0.01 |
| Pets no (n = 51), yes (n = 43)* | 0.47 | 0.40 | 7.9 x 10^-4^ | 7.2 x 10^-3^ |
| **Saliva only:** |  |  |  |  |
| CoNS; -ve (n = 35), +ve (n = 62) | 2 x 10^-3^ | 0.02 | na | na |
| *H. influenzae*: -ve (n = 74), +ve (n = 23) | 0.40 | 0.74 | na | na |
| *S. pneumoniae*: -ve (n = 79), +ve (n = 18) | 0.73 | 0.88 | na | na |
| *M. catarrhalis*: -ve (n = 86), +ve (n = 11) | 0.14 | 0.19 | na | na |

**Table S3. Alpha diversity of covariates in oral and gut microbes from all study participants.**

*some data was lost due to pandemic distruption.


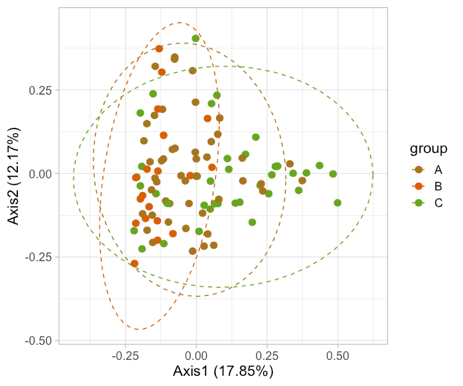

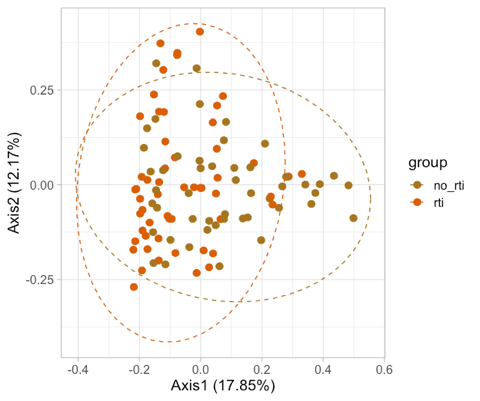

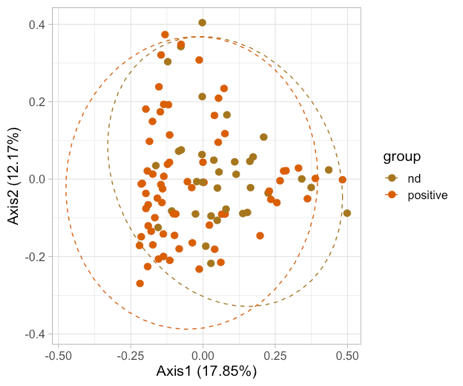

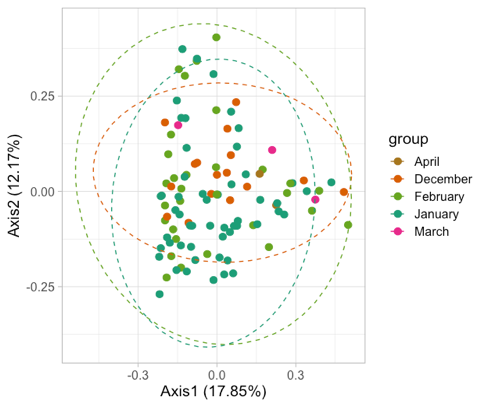


Oral


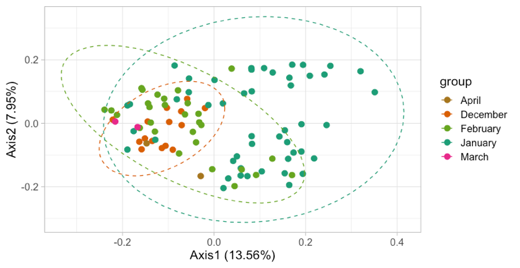

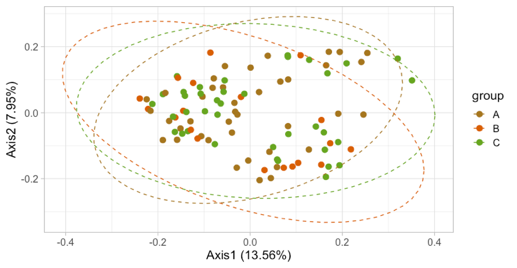

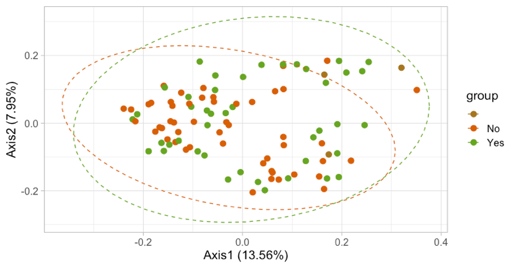

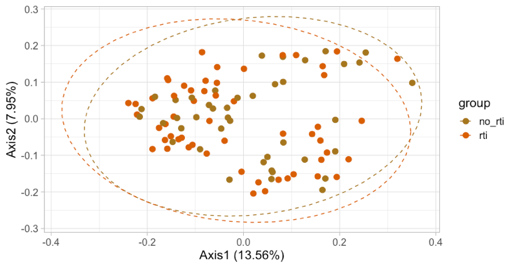


Gut

A

B

C

D

A

B

C

D

**Figure S2. Principal Coordinate Analysis (PCoA) plots based on Bray-Curtis distances for oral-gut microbes.**

PERMANOVA tests for oral microbial covariates were, (A) months (*p* = 0.013), (B) time points (*p* = 0.001), (C) CoNS carriage (*p* = 0.002) and (D) no-RTI vs RTI-S (*p* = 0.001) and gut microbe covariates were (A) months (*p* = 0.001), (B) time points (*p* = 0.893), (C) pet ownership (*p* = 0.007) and (D) no-RTI vs RTI-S (*p* = 0.139). Abbreviations: RTI symptoms (RTI-S) and no symptoms (no-RTI), coagulase-negative Staphylococcus (CoNS), nr = not recorded.


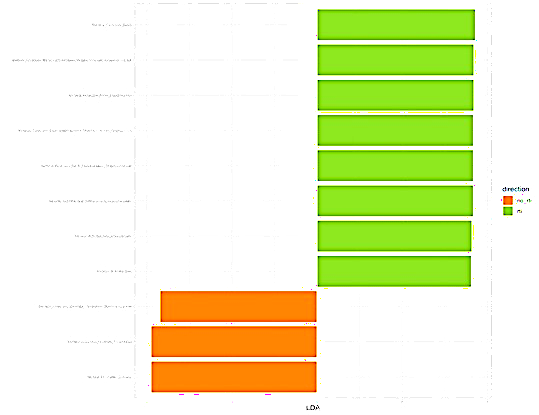


Bacili

*Streptococcus sp.*

Lactobacillus

*Streptococcus sp.*

Streptococcaceae

Actinomycetales

Actinobacteria

Actinobacteria

Ruminococcaceae

Clostridiales

Clostridia

4

2

0

-2

-4

LDA SCORE (log 10)


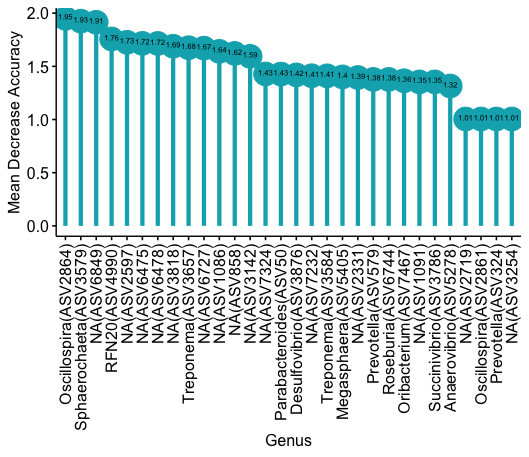


**Figure S3A. Oral microbial biomarkers from participants with RTI symptoms compared those with no symptoms in samples collected at end of the study.**

LEfSe generated LDA scores (log 10) (top) and ranking of the MDA values in random forest model (bottom).


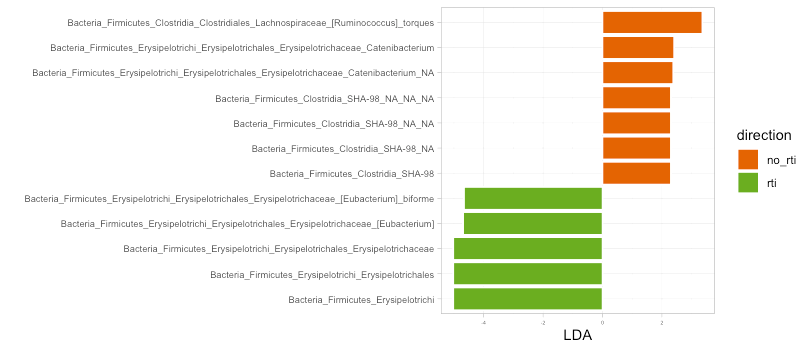


*Ruminococcus torques*

Catenibacterium

Catenibacterium sp.

Clostridia

*Clostridia sp.*

*Eubacterium biforme*

Eubacterium

Eubacterium

Erysipelotrichaceae

Erysipelotrichales

*Coprobacillus*

*Eubacterium sp.*

LDA SCORE (log 10)

-2

0

2

4

-4


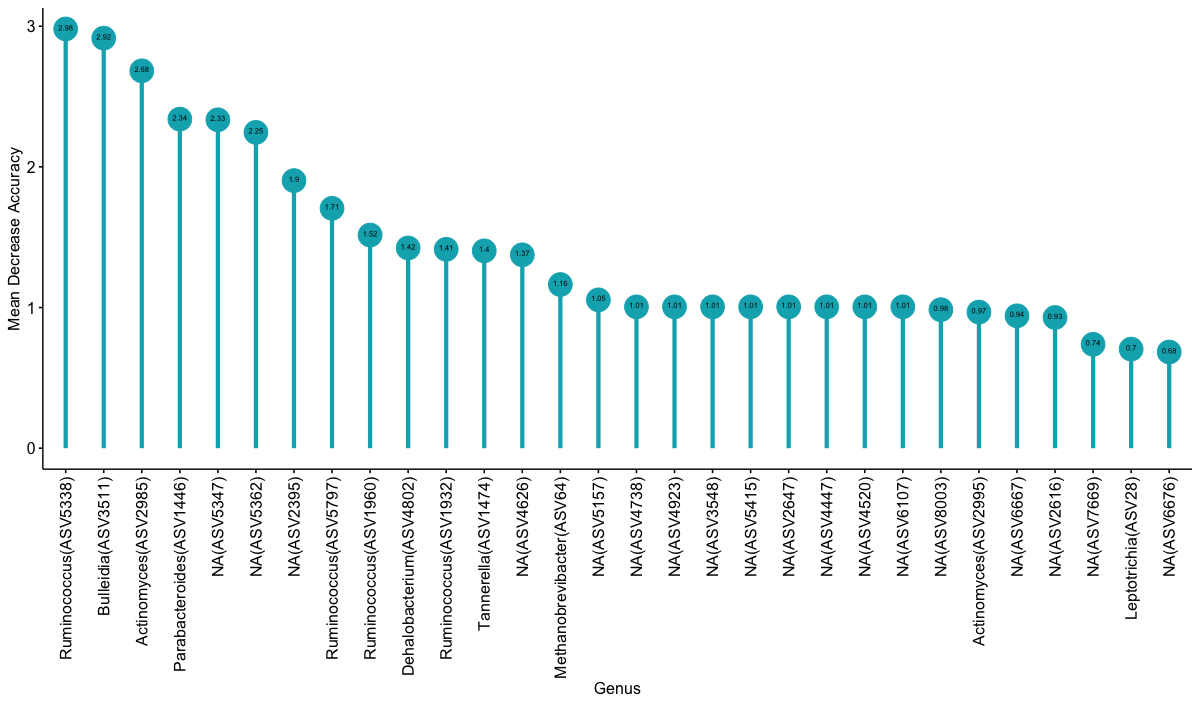


**Figure S3B. Gut microbial biomarkers from participants with RTI symptoms compared those with no symptoms in samples collected at end of the study.**

LEfSe generated LDA scores (log 10) (top) and ranking of the MDA values in random forest model (bottom).
